# Socio-spatial differences in overweight and obesity among preschoolers: An overview using data from the school entry examination

**DOI:** 10.25646/14294

**Published:** 2026-07-15

**Authors:** Charlotte Kühnelt, Anne Starker, Noreen Augel, Christin Dilger, Gabriele Morlock, Kristin Mühlenbruch, Sylke Oberwöhrmann, Jörg Rech, Steffen Schüle, Almuth Spröwitz, Anja Schienkiewitz

**Affiliations:** 1 Robert Koch Institute, Department of Epidemiology and Health Monitoring, Berlin, Germany; 2 Ministry of Science and Health of the State of Rhineland-Palatinate, Mainz, Germany; 3 Ministry of Social Affairs, Labor, and Health Baden-Württemberg, Stuttgart, Germany; 4 Bavarian Health and Food Safety Authority, Oberschleißheim, Germany; 5 Ministry of Health and Social Affairs of the State of Brandenburg, Potsdam, Germany; 6 Senate Department for Higher Education and Research, Health, and Long-Term Care, Berlin, Germany; 7 Ministry of Labor, Social Affairs, Women, and Health, Saarbrücken, Germany; 8 The Senator for Health, Women, and Consumer Protection, Bremen, Germany; 9 State Office for Consumer Protection of Saxony-Anhalt, Halle, Germany

**Keywords:** AdiRaum 20, Children, Overweight, Obesity, Prevalence, Socioeconomic deprivation, Urbanisation

## Abstract

**Background:**

School entry examinations (SEE) conducted by the federal states provide measurement data on the height and weight of preschool-aged children. Prevalence rates for overweight and obesity are available for Health Reporting and can be linked to other factors at the aggregate level. This article describes the prevalence of overweight and obesity among preschool children based on aggregated data from SEE, differentiated by two socio-spatial indicators.

**Method:**

In the AdiRaum 2.0 project, aggregated SEE data on overweight and obesity from the 2006 – 2024 school entry years were compiled from 13 federal states (where available). As an example, the prevalence of overweight and obesity among children starting school in 2019 was linked to the degree of urbanisation and socioeconomic deprivation.

**Results:**

Data from 4,269,299 children aged 4 to 7 years were collected. Between 2006 – 2024, the prevalence of overweight (including obesity) ranged from 8.6 % to 13.4 %, and the prevalence of obesity ranged from 3.7 % to 5.9 %. In 2019, children from districts with high socioeconomic deprivation were more frequently affected by obesity than children from other districts.

**Conclusions:**

The analyses confirm that high socioeconomic deprivation is a key risk factor for overweight and obesity already in childhood. Aggregated SEE data on preschool-aged children are suitable for this analysis. Prevention efforts should address and reduce health inequalities in childhood at the environmental level.

**Key messages**

► In AdiRaum 2.0, aggregated data on overweight and obesity from school entry examinations (SEE) across several federal states were compiled and analyzed.
► At the aggregate level, SEE data are suitable for illustrating regional differences in prevalence.
► Between 2006 and 2024, 8.6 % to 13.4 % of preschool children were affected by overweight (including obesity) and 3.7 % to 5.9 % by obesity.
► The higher the socioeconomic deprivation in the district, the higher the prevalence of overweight and obesity.
► Preventive measures should also be aimed at reducing health inequalities.

**AdiRaum dashboard**

The AdiRaum dashboard provides a visual and easily accessible overview of the prevalence of overweight and obesity among preschool-aged children in Germany’s districts and independent cities. It is based on aggregated data from the School Entry Examination (SEE) in nine federal states participating in the project that have consented to the publication of their data: Baden-Württemberg, Bavaria, Berlin, Brandenburg, Bremen, Hesse, Saarland, Saxony-Anhalt, and Schleswig-Holstein. Additionally, socio-spatial information from other public data sources at the district level is incorporated.

The prevalence rates of overweight and obesity are presented according to characteristics from the SEE, such as age and gender, as well as according to socio-spatial factors, such as the residential structure of district or the degree of socioeconomic deprivation.

Furthermore, the dashboard provides information on the methodology used in the AdiRaum 2.0 project and an overview of dashboards and reports from the federal states’ Health Reporting (GBE).

The underlying data will be updated regularly and expanded to include additional states. In the spirit of open data, it is published on GitHub and Zenodo.

**Link to the dashboard:** https://public.data.rki.de/t/ public/views/AdiRaum/Dashboard

**Infobox Web portal of Federal Health Reporting**

The website www.gbe.rki.de/EN of Federal Health Reporting at the Robert Koch Institute (RKI) provides reliable information on the health situation of the population in Germany: timely, transparent and easily accessible. The focus is on noncommunicable diseases such as diabetes mellitus, cardiovascular diseases, cancer and mental disorders. It also presents factors that have an influence on health, such as health behavior or social determinants. In addition, the web portal provides information on health care and contextual factors, such as tobacco control measures, which also influence the health of the population.

The website currently includes 78 indicators based on the health studies conducted by the RKI and other data sources, which are interactively visualized and contextualised in short texts. The data is published as open data on GitHub and Zenodo. In addition, the website provides access to all publications of the Journal of Health Monitoring as well as to other scientific articles from the RKI that are related to the topics on the website. The content is continuously expanded.

Further information on the topic of this article can be found on the web portal at: www.gbe.rki.de/obesity-overweight-preschoolchildren

## 1. Introduction

Overweight and obesity in children and adolescents are associated with adverse long-term health consequences and can increase the risk of developing chronic diseases, such as type 2 diabetes and high blood pressure, later in life [1–3]. Results from the second wave (2014 – 2017) of the ‘German Health Interview and Examination Survey for Children and Adolescents’ (KiGGS) show that among children aged three to six years, the prevalence of overweight (including obesity) was 9 % and that of obesity was 2 % [4]. These results are based on measurement data. From the KiGGS baseline survey (2003 – 2006) to KiGGS Wave 2, the prevalence of overweight and obesity has not changed significantly. However, during the COVID-19 pandemic, national and international studies have observed an increase in body mass index (BMI) and in the prevalence of overweight and obesity among children and adolescents [5–8]. 

Currently, various studies in Germany have provided survey and measurement data on the height and weight of children and adolescents in different age groups [9–11]. Since information provided by parents or self-reported data from children and adolescents are subject to potential biases – such as underestimating weight or overestimating height – the calculated BMI and the prevalence of overweight and obesity may be lower than the actual values [12–15]. To perform valid prevalence estimates, current measurement data are also required. Current population-based data on overweight and obesity in childhood and adolescence, based on measurement data, were collected in a further wave (2023 – 2025) of the Motorik Monitoring 2.0-Study (MoMo 2.0) and are currently being analyzed [16].

For preschool-aged children in Germany, a health and developmental assessment is part of the mandatory School Entry Examination (SEE), during which height and weight are measured. The goal of the examination is to identify developmental abnormalities or disorders before the start of school and, if necessary, to initiate support. The SEE can be assumed to represent a complete survey of the children starting school in the respective year. Individual data from the SEE, e.g., on overweight and obesity, are available in the districts and federal states and can be aggregated at various levels for the Health Reporting (German: Gesundheitsberichterstattung, GBE). 

This aggregated, local-level data can be linked to socio- spatial indicators, such as socioeconomic deprivation at the district level. National and international analyses show that the prevalence of obesity among children is higher in districts with high socioeconomic deprivation than in districts with low deprivation [5, 17, 18]. The degree of urbanisation can also influence the prevalence of overweight and obesity. Among adults, rural areas of Germany have higher rates of obesity than urban areas [19]. For preschool-aged children, this association has not yet been examined at the district level. The local-level analysis of SEE data can help to illustrate and quantify this association.

As a regular cross-sectional survey, the SEE is also suitable for describing trends over time: For example, the GBE of Lower Saxony reported an increase in the prevalence of overweight and obesity at the onset of the COVID-19 pandemic, based on SEE data [20, 21]. Since 2022, however, the proportion of children who are overweight or obese has decreased again and approached the expected value of the trend calculation [22]. This trend can also be observed, among other places, in the SEE data for the city of Bremen and the state of Brandenburg [8, 23]. As part of the AdiRaum 2.0 project, SEE data aggregated at the district level was compiled for the Federal Health Reporting at the Robert Koch Institute (RKI) and presented in a dashboard.

The aim of this paper is to describe the prevalence of overweight (including obesity) and obesity in preschool-aged children based on aggregated SEE data from 13 federal states. To illustrate socio-spatial differences, the prevalence of overweight and obesity for the 2019 school entry cohort (calendar year of school entry) is also presented, stratified by degree of urbanisation and socioeconomic deprivation. 

## 2. Method

### 2.1 Data source

As part of the AdiRaum and AdiRaum 2.0 projects, SEE data from 13 federal states were compiled by August 2025 (total dataset). The states included were Baden-Württemberg, Bavaria, Berlin, Brandenburg, Bremen, Hamburg, Hesse, Lower Saxony, North Rhine-Westphalia, Rhineland-Palatinate, Saxony, Saxony-Anhalt, and Schleswig-Holstein. These states provided aggregated data on children aged four to seven who started school between 2006 and 2024 and were examined as part of the SEE. The data provided covered 95 % to 99 % of the children starting school in the respective school entry year in the participating federal states. The number of states that submitted data to the RKI varied between two and 13 states depending on the year, so that the sample size fluctuated between 28,223 children in 2021 and 615,086 children in 2019 (Table 1). 

For each school entry year, the RKI received the number of children examined (only children with complete data on gender, age, height and weight were included) and the number of children with overweight (including obesity) or obesity per district, stratified by gender and age [24]. Based on this data, the prevalence of overweight and obesity was calculated for each district, differentiated by school entry year, gender and age. When case numbers were low, the midpoint was imputed – taking state-specific confidentiality rules (e.g., 0 < n ≤ 5) into account – to reduce the risk of re-identification. Children with the gender entry ‘diverse’ or a missing entry could not be included in the analyses, because case numbers were low and no age-specific percentiles were available.

The stratified analyses of the prevalence of overweight and obesity by socio-spatial indicators were based on data from the 2019 school entry year, as data for this year were available from 12 (of 13) federal states.

### 2.2 Classification of overweight and obesity

Before the data was transmitted to the RKI, the children’s BMI was calculated in the federal states based on individual height and weight measurements. Body weight was classified in relation to height according to age (on a monthly basis) and sex, using the Kromeyer-Hauschild population-based reference system: If a child’s BMI exceeded certain reference values, the child was classified as overweight or obese (overweight including obesity: BMI > 90th percentile; obesity: BMI > 97th percentile) [25].

### 2.3 Socio-spatial indicators

To enable a differentiated presentation of prevalence rates according to socio-spatial stratification characteristics, additional indicators were identified in other data sources that allow for a comparison of districts or that are relevant to childhood obesity [24]. This was based, among other things, on the AdiMon project, where a systematic literature review was conducted and indicators relevant to the development of childhood obesity were identified [26]. In this study, two of these indicators are discussed in relation to overweight and obesity in the 2019 school entry year: the degree of urbanisation and the German Index of Socioeconomic Deprivation (GISD). 

#### Degree of urbanisation

For the degree of urbanisation at the district level an indicator defined by the Federal Institute for Research on Building, Urban Affairs and Spatial Development (BBSR) was used. It is based on the population density of the district, with and without consideration of large and medium-sized cities, and the proportion of the population living in large and medium-sized cities [27]. Based on these characteristics, each district was assigned one of four categories for the year 2019: ‘sparsely populated rural district’, ‘rural district’, ‘urban district’ and ‘independent large city’. In Bavaria, 19 public health offices are responsible for both a rural district and an independent city, which is why data collection and presentation are conducted together there. Since, in these cases, the data is not available separately at the district level, these 38 districts were therefore excluded from the urbanisation category analyses. In this study, the districts of the three city-states (Berlin, Bremen, and Hamburg) were classified as independent large cities.

#### German Index of Socioeconomic Deprivation (GISD)

The GISD is an indicator of the extent of regional socioeconomic disadvantage [28]. The GISD uses administrative data on education, employment, and income at the district level, provided by the BBSR. The following nine indicators are used to calculate the GISD: proportion of employed persons with a college or university degree, proportion of employed persons without a vocational qualification, proportion of school leavers without a diploma, employment and unemployment rates, gross wages and salaries, net household income, debt ratio, and income tax revenue. The GISD score is available for each district as a measure of socioeconomic deprivation and ranges from 0 (lowest deprivation) to 1 (highest deprivation). These values are divided into quintiles on an annual basis, with the lowest quintile representing ‘low’ socioeconomic deprivation and the highest quintile representing ‘high’ socioeconomic deprivation, while the other three quintiles are grouped together as ‘average’ (hereinafter referred to as ‘medium’). For a detailed description of the methodology, see Michalski et al. [28]. For the year 2019, each district received a GISD score and was assigned to one of the three categories based on the current GISD data (January 31, 2025). For the districts of Bavaria in which a public health office is responsible for both a rural district and an independent city, no GISD was assigned; therefore, these 38 districts were not included in the analyses by GISD. Since not all parameters necessary for calculating the GISD at the district level are currently available for the city-states, the districts of the city-states were assigned the GISD of the respective city-state.

### 2.4 Analyses

Descriptive statistics were calculated to characterize the population of the total dataset and to analyze the distribution of overweight and obesity prevalence across the selected indicators. The relationship between the GISD score and obesity prevalence was depicted in a scatter plot. Each district and each independent city was represented by a point, and a linear regression line showed the trend in the relationship between the two variables. Additionally, the categorical GISD was used to examine group differences in obesity prevalence. The correlation between the GISD and the district type was examined using Spearman’s rank correlation. To examine differences in the prevalence of overweight and obesity between urbanisation and GISD categories, mixed-effects Poisson regressions were performed, which account for the hierarchical data structure of the dataset as well as fixed and random effects. The model accounted for differences in obesity rates between districts as well as individual differences. A statistically significant difference between the categories of urbanisation and the GISD was assumed if the corresponding p-value was less than 0.01 (**) or 0.001 (***). The analyses were performed in STATA/SE 17. 

## 3. Results 

### 3.1 Study population and prevalences

The project’s total dataset comprised 4,269,299 children aged four to seven who started school between 2006 and 2024. Of these, 48.5 % were girls. At the time of the SEE, most of the children was five years old (55.3 %) or six years old (33.3 %), 10.9 % were four years old, and only 0.5 % were seven years old (detailed data not shown). 

Table 1 shows that not all federal states reported data to the RKI for the entire period from 2006 to 2024. Data availability varied significantly among federal states and was highest for the school entry years 2015 through 2019. During this period, the prevalence of overweight and obesity remained largely stable. During the COVID-19 pandemic (2020 – 2022 school entry years), data availability was limited to two to five federal states where the SEE was fully conducted. During this period, prevalence rates tended to be higher than in the years 2015 – 2019. By 2024, prevalence rates had declined to pre-pandemic levels or were even lower. In the overall dataset, the prevalence of overweight (including obesity) ranged from 8.6 % in 2024 to 13.4 % in 2021, with an average of 10.3 %. The average prevalence of obesity was 4.3 %, ranging from 3.7 % in 2014 and 2024 to 5.9 % in 2021. The prevalences of overweight and obesity varied only slightly between girls and boys (0.1 percentage points for overweight and 0.3 percentage points for obesity). 

The dataset for the analyses of the 2019 school entry cohort included 615,086 children. Like in the overall data set, 48.5 % of the children were girls. 61.5 % were five years old and 30.6 % were six years old. 7.7 % were four years old and only 0.2 % were seven years old. In 2019, the prevalence of overweight (including obesity) was 10.0 %, and 4.2 % of the children were obese. The prevalences differed only slightly between girls and boys (0.2 percentage points). Since the study focused on describing the prevalence of overweight and obesity at local level by socio-spatial indicators, and the authors were not aware of any differences in these associations between girls and boys in this age group, the analyses presented below cover the entire sample. However, prevalence rates were determined using gender-specific percentiles. 

### 3.2 Distribution of overweight (including obesity) and obesity by the degree of urbanisation

The majority of children in the 2019 school entry cohort lived in urban districts (39.8 %, n = 233,295) or independent large cities (33.2 %, n =194,399). 15.5 % lived in rural districts (n = 90,698) and 11.5 % in sparsely populated rural districts (n = 67,573). 29,121 children lived in districts that could not be assigned to an urbanisation category (see Method).

**Figure 1:**
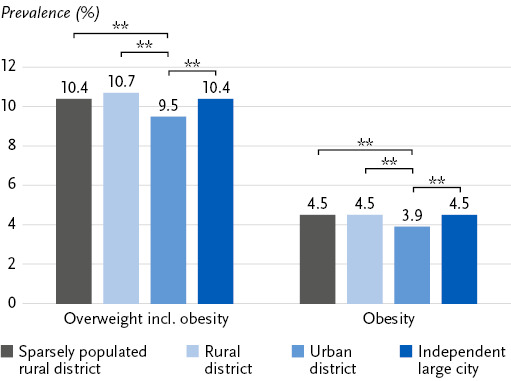
Prevalence of overweight (including obesity) and obesity (%) in the study population of the 2019 school entry cohort, by urbanisation category (12 federal states; n = 585,9651) among 4- to 7-year-olds (as of 08/2025). Source: School entry examinations ^1^Discrepancies in the number of cases in Table 1 result from missing values in the urbanisation category (missing values: n = 29,121); ^**^: p < 0.01

Differences in the prevalence of overweight and obesity were observed by urbanisation category (Figure 1). The lowest rates were found in urban districts (overweight including obesity: 9.5 %; obesity: 3.9 %). The prevalence rates there were statistically significantly lower than in the other categories; however, the differences amounted to only 0.9 –1.2 percentage points for overweight including obesity and 0.6 percentage points for obesity. The prevalence rates of the other categories did not differ significantly from one another. 

### 3.3 Distribution of overweight (including obesity) and obesity by socioeconomic deprivation

The majority of children in the 2019 school entry cohort lived in districts with medium deprivation (64.4 %, n = 377,616), 12.8 % in districts with high deprivation (n = 74,956), and 22.8 % in districts with low deprivation (n =133,393). 29,121 children lived in districts to which no GISD score could be assigned (see Method). 

**Figure 2:**
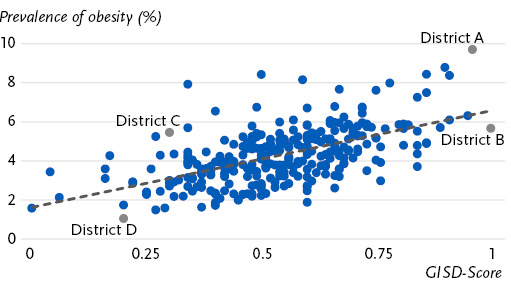
Prevalence of obesity (%) in the study population of the 2019 school entry cohort across districts and independent cities by GISD score (12 federal states; n = 585,9651) among 4- to 7-year-olds (as of 08/2025). Source: School entry examinations ^1^Discrepancies in the number of cases in Table 1 result from missing values in the GISD (missing values: n = 29,121); GISD: German Index of Socioeconomic Deprivation

Figure 2 illustrates the correlation between the prevalence of obesity and the GISD score. At the district level, the regression line showed a positive association between the GISD and the prevalence of obesity: as the GISD score increased, the prevalence of obesity in the districts also increased. This means that as socioeconomic deprivation increased, the proportion of obese children rose. This was also observed for the correlation between overweight and GISD (Supplementary material Figure 1). As an example, the differences in obesity prevalence between four independent cities were highlighted: District A had the highest prevalence of obesity (9.7 %), accompanied by a high GISD score of 0.96. In District B, the prevalence of obesity (6,0 %) was below the expected level despite a similarly high GISD score (1.0). District C showed a similarly high prevalence of obesity (5.5 %) as District B, although the GISD score (0.3) was significantly lower. District D had the lowest prevalence of obesity (1.1 %), although the GISD score (0.2) was similarly high as in District C.

**Figure 3:**
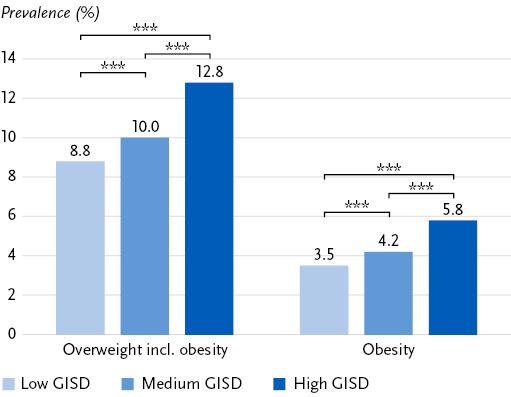
Prevalence of overweight (including obesity) and obesity (%) in the study population of the 2019 school entry cohort by GISD category School entry examinations ^1^Discrepancies in the number of cases in Table 1 result from missing values in the GISD (missing values: n = 29,121); ^***^: p < 0.001; GISD: German Index of Socioeconomic Deprivation

Statistically significant differences in the prevalence of overweight and obesity were also observed between the three GISD categories (Figure 3). A rising gradient from low to high deprivation was evident. The prevalence of overweight (including obesity) was statistically significantly higher in districts with high deprivation (12.8 %) than in districts with low (8.8 %) or medium deprivation (10.0 %). This also applied to the prevalence of obesity: it was 5.8 % in districts with high deprivation and statistically significantly higher compared to districts with low (3.5 %) and medium (4.2 %) deprivation. 

### 3.4 Distribution of overweight (including obesity) and obesity by degree of urbanisation and socioeconomic deprivation

The dataset allowed for the combination of different socio-spatial variables at the district level, so that the distribution of children entering school in 2019 by urbanisation and GISD categories is presented below (Table 2). More than a quarter of the children entering school in 2019 lived in urban districts with medium socioeconomic deprivation (28.6 %). The smallest proportion of children lived in sparsely populated districts with low deprivation (1.1 %) and urban districts with high deprivation (1.3 %). Children in districts with low deprivation lived predominantly in urban districts (9.8 %) or independent large cities (9.9 %), and children in districts with high deprivation lived mostly in independent large cities (6.3 %). A weak, but statistically significant negative correlation was observed between the GISD and the degree of urbanisation (p = − 0.14; p < 0.001).

**Figure 4:**
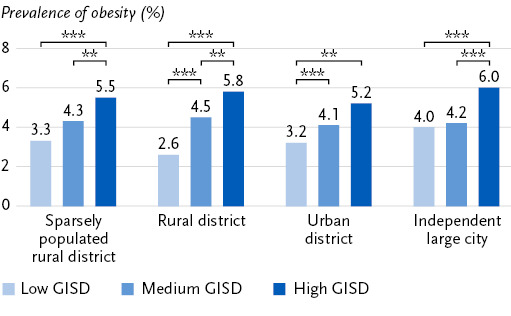
Obesity prevalence (%) in the study population for the 2019 school entry cohort by urbanisation and GISD categories (12 federal states; n = 585,9651) among 4- to 7-year-olds (as of 08/25). Source: School entry examinations ^1^Discrepancies in the number of cases in Table 1 result from missing values in the urbanisation and GISD categories (missing values: n = 29,121); ^**^: p < 0.01; ^***^: p < 0.001; GISD: German Index of Socioeconomic Deprivation

Within each urbanisation category, a gradient in obesity prevalence was observed from low to high socioeconomic deprivation, which was largely statistically significant (Figure 4). The same pattern was observed for overweight (Supplementary material Figure 2). The prevalence of obesity was lowest in rural districts with low deprivation (2.6 %) and highest in independent large cities with high deprivation (6.0 %). At both low (4.0 %) and high (6.0 %) deprivation levels, independent large cities had higher obesity prevalences than other urbanisation categories with the same GISD category.

## 4. Discussion

This study describes the prevalence of overweight and obesity among preschool children from 2006 to 2024 based on data from school entry examinations in 13 federal states. The prevalence of overweight and obesity remained largely stable over time, and the prevalence of obesity was higher than in comparable studies. In the 2019 school entry year, a strong socioeconomic gradient was observed. Linking the data with two socio-spatial indicators highlighted the data’s potential for analyzing and describing prevalence rates and trends.

### 4.1 Contextualisation

The prevalence of overweight (including obesity) among 4- to 7-year-olds in the overall dataset (2006 – 2024) ranged from 8.6 % (2024) to 13.4 % (2021). The results for the period 2014 – 2017 (9.9 % –10.1 %) were of a similar magnitude to the prevalence of 9.0 % (95 % CI: 6.7 –12.1) among 3- to 6-year-olds in KiGGS Wave 2 during the same period [4]. In contrast, data from the third wave (2018 – 2022) of the MoMo- Study showed lower prevalence rates of overweight (7.3 %) among children aged four to six years [29]. It is possible that the MoMo-Study, with its focus on physical fitness and activity behavior, exhibits a selection bias, resulting in children who are overweight or obese being underrepresented in the sample.

In the SEE data, the prevalence of obesity ranged from 3.7 % (2014 and 2024) to 5.9 % (2021) and from 3.7 % to 4.1 % in 2014 – 2017. In all years, it thus remained above the expected value of 3 % and was higher than the prevalence of 2.0 % reported in KiGGS Wave 2 for 3- to 6-year-olds (95 % CI: 1.1 – 3.6) [4]. Differences in prevalence rates compared to the KiGGS study may be attributed to the underlying study populations: On the one hand, the SEE is a complete survey; on the other hand, the children at the time of the SEE were slightly older than the population in the KiGGS study. Even within this age group, an increase in obesity prevalence with age has been observed in the literature [30]. Thus, in KiGGS Wave 2, already 15.5 % of children aged seven to eleven were overweight and 5.8 % were obese. In the present study, prevalence rates differed only slightly between girls and boys. Due to developmental factors, differences in prevalence between the sexes are not expected until the onset of puberty. 

#### COVID-19 pandemic

Another reason for the discrepancy in overall prevalence rates compared to results from other studies may be that data from the COVID-19 pandemic period were included in the calculation. The prevalence of overweight and obesity prior to the COVID-19 pandemic (2015 – 2020) was largely stable both in this study (overweight prevalence (including obesity) of 9.9 % to 10.5 % and obesity prevalence of 4.0 % to 4.4 %) and in comparable data on children aged four to five years from the National Child Measurement Programme (NCMP) in the United Kingdom, but rose significantly during the pandemic [21, 31]. The COVID-19 pandemic impacted the implementation of the SEE, which is why, for example, in some districts not all children could be offered a SEE, and thus a complete survey is not available. Consequently, the total dataset contained data for the school entry years of 2020, 2021, and 2022 from only a few federal states. This must be taken into account when interpreting the results. The data available for this period came predominantly from federal states whose obesity prevalence was already above the average of 4.3 % prior to the COVID-19 pandemic. Data availability increased again after the COVID-19 pandemic, and prevalence rates returned to pre-pandemic levels, even falling below them in 2024 to 8.6 % for overweight (including obesity) and 3.7 % for obesity. However, reliable reporting on the trend requires SEE data from more federal states before, during, and after the pandemic. Separate analyses of SEE data from Lower Saxony also showed that prevalence rates returned toward pre-pandemic levels after 2022, in line with the 2015 – 2019 trend [22]. A similar trend was observed in the SEE data from Brandenburg [8]. Continuously collected data in the United Kingdom also showed that prevalence rates among preschool children returned to pre-pandemic levels following the COVID-19 pandemic [5]. In Denmark, while the prevalence of overweight among first-graders declined after the COVID-19 pandemic, the prevalence of obesity remained elevated [32]. The European Childhood Obesity Surveillance Initiative (COSI), which examined children aged six to nine, revealed a mixed picture across various European countries, with obesity prevalence in some countries remaining higher after the pandemic (2022 – 2024) than before (2018 – 2020) [33]. 

#### Overweight (including obesity) and obesity by degree of urbanisation 

According to the available analyses, in 2019 73 % of children lived in urban districts or independent large cities (see Table 2). This is broadly consistent with the general population, of which 77 % lived in urban areas [34]. Although the SEE data did not show a clear gradient in obesity prevalence depending on the degree of urbanisation, they did reveal statistically significant differences in the prevalence of overweight and obesity between urban and rural districts: the prevalence was significantly lower in urban districts than in rural districts and independent large cities. Separate analyses using SEE data from Brandenburg even revealed differences within the districts: In the Berlin suburbs, prevalence rates were lower than in the wider metropolitan area [8]. It has been observed that in rural regions, contextual factors in particular contribute to the development of obesity in childhood and adolescence, e.g., a lack of sidewalks and public transportation as well as long distances to recreational facilities [35]. 

#### Overweight (including obesity) and obesity by socioeconomic deprivation 

The prevalence of overweight and obesity among children varied according to the degree of deprivation: an increase in the GISD score was associated with an increase in prevalence. Children living in districts with high deprivation had significantly higher prevalence rates than those with low or medium deprivation. Analyses using SEE data from Düsseldorf, which took into account, among other factors, the level of deprivation in the residential environment, reached the same conclusions: Children living in the most deprived neighborhoods were twice as likely to be obese as those living in the least deprived neighborhoods [17]. In studies using SEE data from Aachen and Munich, children in neighborhoods with low or medium socioeconomic status (SES) were also at increased risk of being overweight, even when the family had a high level of education [18, 36]. In Bremen, during the COVID-19 pandemic, children in districts with a low social index were significantly more affected by the increase in the prevalence of overweight and obesity than those living in districts with a high social index [23]. In the United Kingdom as well, preschool children in highly deprived areas were twice as likely to be affected by obesity as children in the least deprived areas [5]. The reasons for these differences are diverse and complex; among other factors, limited access to healthy food and few opportunities for physical activity (parks, playgrounds, active transportation) have been described in deprived neighborhoods [17, 37]. Those aspects are discussed in the context of so-called obesogenic environments, which describe environmental factors that promote the development of overweight [38]. The KiGGS study provided information on the children’s individual SES, which can also be used to examine the influence of socioeconomic determinants: children and adolescents with low SES were four times more likely to be obese than those with high SES [30]. Social disparities in the prevalence of overweight among children and adolescents have increased over time [39]. Data from the SEE in Brandenburg and Baden-Württemberg also showed that children with low socioeconomic status are more frequently affected by overweight or obesity than children with high socioeconomic status [8, 40]. The results presented at the aggregate level corroborate the findings based on individual data and confirm that poverty is an important risk factor for obesity even in childhood [41]. 

#### Overweight (including obesity) and obesity by degree of urbanisation and socioeconomic deprivation

Differences were observed among the district types within the GISD categories. In particular, a clear increase in prevalence was observed across all urbanisation categories as the level of socioeconomic deprivation increased, indicating the significant relevance of socioeconomic factors. The international literature on the subject is heterogeneous. A U.S. study showed significantly higher obesity rates in rural areas for children aged three to four from low-income families [42]. In contrast, data from the NCMP, showed for children in England that, on the one hand, family income is the most significant predictor of obesity and, on the other hand, that the association between income and obesity is stronger in urban areas than in rural areas [43]. The results underscore the importance of financial poverty regardless of the place of residence. Targeted analyses are needed to better understand regional differences and incorporate them into prevention strategies. 

### 4.2 Strengths and limitations

For the first time, SEE data on overweight and obesity from several federal states were aggregated at the district level and a descriptive analysis was conducted. Since participation in the SEE is mandatory for school-age children in Germany, the dataset was based on a complete survey of the reported school entry years from the participating states. For height and weight, measurement data collected according to comparable guidelines were available, which are more accurate than self-reported data [14]. Because the SEE is conducted annually, time series are generally available that can be used for the regular monitoring of overweight and obesity in the preschool-aged group. At the level of federal states, SEE data are published, for example, in dashboards [44–51].

When interpreting the results, it is important to take into account the limitations of the SEE as a data source: due to Germany’s federal structure, the timing of the SEE varies between the states [52]. The SEE is primarily conducted during the final year of kindergarten; however, in some states, children are assessed in their penultimate year of kindergarten. Additionally, the cut-off dates for school enrollment vary by several months across the federal states. Adjustments to the cut-off date or the timing of the SEE may also occur, leading to changes in the age structure within a state over time [53, 54]. The age composition of the children included in a given year can lead to different prevalence estimates for overweight and obesity across the federal states. In cross-state comparisons, lower prevalence rates may be attributable to a younger age structure at the time of the examination. However, the consistent use of Kromeyer-Hauschild reference values accurate to the age in months improves the precision of the estimator. A look at the reference curves shows that growth and the ratio of body weight to height are particularly dynamic between the ages of five and seven. 

In the overall dataset, the number of federal states varied across school entry years, which could also explain differences in prevalence that can only be reliably assessed if data from the same federal states are available over time. For data protection reasons, state confidentiality rules were applied for small case numbers. To prevent relevant information from getting lost for the analyses, imputations were performed. In some federal states, certain age groups were completely excluded from the analyses due to low case numbers. Aggregated data at the level of districts and independent cities was used, for which structural characteristics at district level were available from public databases. It must be noted that no conclusions about individuals can be drawn from aggregated data (so-called ecological fallacy). 

The analyses were conducted using the 2019 school-entry cohort as an example. Since the GISD was not available for the districts of the city-states at the time of the data analysis, the analyses could not account for socioeconomic differences within the city-states and differences may not have been fully captured. Although children from districts with a high GISD were slightly underrepresented in the study population compared to the general population, this did not constitute a selection bias, as the data were derived from a complete survey of the underlying federal states. This limitation could be mitigated in the future by including additional federal states. A formal test for differences in the association between obesity and the GISD across urbanisation categories was not conducted, but will be the subject of future analyses. In principle, no causal conclusions can be drawn from the results of the analysis using aggregated data. In this study, the district level was chosen as the local unit. However, this can only partially reflect the children’s actual living environment. Therefore, further analyses are necessary, e.g., at the municipal level, to help identify regional factors that are not sufficiently differentiated at the district level. When comparing results with those from other school entry years, it should be noted that the data are cross-sectional and that the composition of the individual school entry cohorts varies from year to year. However, a sensitivity analysis using data from the 2015 and 2018 school entry years confirmed the results of the present analyses for 2019. 

### 4.3 Conclusion

Aggregated SEE data is a suitable data source for describing the prevalence of overweight and obesity among preschool-aged children. When combined with contextual factors, they can be used to illustrate socio-spatial differences in prevalence rates within this age group. The analyses conducted using SEE data aggregated at the district level confirmed what has already been widely described at the individual level: poverty is a significant risk factor for the development of overweight and obesity even in childhood. The higher the degree of socioeconomic deprivation in the districts, the higher the prevalence of obesity, regardless of the degree of urbanisation. A central goal of preventive measures should therefore be to reduce health inequalities in childhood at the environmental level in order to promote healthy development. 

## Figures and Tables

**Table 1:** Total dataset and prevalence (%) of overweight (including obesity) and obesity by gender in the 2006 – 2024 school entry years among 4- to 7-year-olds (as of 08/2025). Source: School entry examinations

**School entry year**	**Number of federal states (n)**	**Number of children (n)**	**Prevalence of overweight (including obesity; %)**			**Prevalence of obesity (%)**		
			**Total**	**Girls**	**Boys**	**Total**	**Girls**	**Boys**
2006	2	80,938	12.4	12.1	12.7	5.1	4.8	5.4
2007	2	77,509	12.4	12.2	12.6	5.1	4.8	5.4
2008	2	63,221	11.7	11.5	11.9	4.7	4.4	5.0
2009	2	75,197	11.6	11.5	11.7	4.6	4.4	4.8
2010	2	68,729	12.5	12.5	12.4	5.1	5.0	5.2
2011	3	145,012	10.5	10.4	10.5	4.0	3.9	4.2
2012	3	148,355	10.7	10.5	10.8	4.2	4.0	4.5
2013	3	150,996	10.1	10.1	10.2	3.9	3.8	4.0
2014	3	152,734	9.9	9.9	9.8	3.7	3.6	3.8
2015	10	544,092	9.9	10.0	9.7	4.0	3.9	4.0
2016	9	462,433	10.1	10.1	10.0	4.1	4.0	4.2
2017	9	473,370	10.0	10.1	9.8	4.1	4.0	4.2
2018	10	484,941	10.5	10.5	10.4	4.4	4.3	4.5
2019	12	615,086	10.0	10.1	9.9	4.2	4.1	4.3
2020	4	132,801	9.6	9.6	9.6	3.8	3.8	3.9
2021	2	28,223	13.4	13.3	13.5	5.9	5.8	6.0
2022	5	152,709	11.7	11.8	11.7	5.4	5.3	5.6
2023	7	231,081	10.7	10.7	10.7	4.8	4.7	4.9
2024	4	181,872	8.6	8.8	8.5	3.7	3.7	3.7
**Total**	**13**	**4** **,** **269** **,** **299**	**10** **.** **3**	**10** **.** **4**	**10** **.** **3**	**4** **.** **3**	**4** **.** **1**	**4** **.** **4**

**Table 2:** Distribution of the study population for the 2019 school entry cohort by urbanisation and GISD categories (12 federal states; n = 585,9651) among 4- to 7-year-olds (as of 08/2025). Source: School entry examinations

**Urbanisation category**	**GISD category**			**Total**
	**low**	**medium**	**high**	
Sparsely populated rural district	6,575 (1.1 %)	46,704 (8.0 %)	14,294 (2.4 %)	67,573 (11.5 %)
Rural district	11,027 (1.9 %)	63,976 (10.9 %)	15,695 (2.7 %)	90,698 (15.5 %)
Urban district	57,583 (9.8 %)	167,836 (28.6 %)	7,876 (1.3 %)	233,295 (39.8 %)
Independent large city	58,208 (9.9 %)	99,100 (16.9 %)	37,091 (6.3 %)	194,399 (33.2 %)
**Total**	**133** **,** **393** ** (** **22** **.** **8** ** %)**	**377** **,** **616** ** (** **64** **.** **4** ** %)**	**74** **,** **956** ** (** **12** **.** **8** ** %)**	**585** **,** **965** ** (** **100** ** %)**

^1^Discrepancies in the number of cases in Table 1 result from missing values in the urbanisation and GISD categories (missing values: n = 29,121); GISD: German Index of Socioeconomic Deprivation
